# Psychometric evaluation of the Adult Eating Behavior Questionnaire and its relationship with body mass index among Chinese university students: a cross-sectional validation study

**DOI:** 10.3389/fpubh.2025.1691302

**Published:** 2025-11-12

**Authors:** Qinyu Yan, Muhammad Waseem Shah, Jin Yang, Da Pan, Guiju Sun

**Affiliations:** Key Laboratory of Environmental Medicine and Engineering of Ministry of Education, Department of Nutrition and Food Hygiene, School of Public Health, Southeast University, Nanjing, China

**Keywords:** adult eating behavior questionnaire, body mass index, Chinese university students, appetitive traits, obesity

## Abstract

**Background:**

Eating behavior critically impacts human health and the development of obesity. This study aimed to validate the Adult Eating Behavior Questionnaire (AEBQ) among Chinese university students and explore the relationship between appetitive traits and body mass index (BMI).

**Methods:**

A total of 546 students from Southeast University completed the Chinese version of the AEBQ and self-reported anthropometric data. Confirmatory factor analysis (CFA) was used to test the factor structure and evaluate model fit, and Spearman’s correlation assessed relationships between appetitive traits and BMI.

**Results:**

The original 8-factor, 35-item model showed a good fit, which improved further after removing the Hunger subscale. All subscales demonstrated strong reliability (*α* and *ω* > 0.70). Females exhibited higher scores in Enjoyment of Food, Emotional Over-eating, Food Responsiveness, and Satiety Responsiveness than males (*p* < 0.05). Food approach traits (except for Hunger) were positively correlated with BMI (*p* < 0.05), whereas food avoidance traits (except for Food Fussiness) were negatively correlated (*p* < 0.05).

**Conclusion:**

The AEBQ is a reliable and valid psychological measurement for assessing appetitive traits in Chinese adults and conducting large-scale studies. Interventions targeting appetite traits provide new insights into weight management and obesity prevention.

## 1 Introduction

Over the past two decades, obesity has reached pandemic levels worldwide, with more than two billion individuals now classified as overweight or obese (1). In China, obesity prevalence has risen rapidly, reaching 17.10% in men and 13.37% in women (2). In epidemiological research, obesity is typically defined using the Body Mass Index (BMI), a widely accepted metric for assessing body fat and associated health risks (3). Many observations suggest that obesity is associated with increased risks of diabetes, cardiovascular disease, and cancer (4), and has thus become a major public health concern (5).

An imbalance between energy intake and expenditure is a key determinant of obesity (6). Eating behavior—an important factor influencing energy intake—plays a critical role in human growth, development, and the onset of chronic conditions such as obesity (7, 8). Appetitive traits describe stable tendencies toward food approach or avoidance, reflecting how individuals respond to internal cues (e.g., hunger, satiety, emotions) and external cues (e.g., food availability or sensory appeal) (9). These traits differ across individuals and are shaped by both genetic and environmental factors (10). Individuals with stronger food-approach traits tend to overeat and gain weight, whereas those with stronger food-avoidance traits tend to maintain a lower BMI (11). In addition, certain appetitive traits seem to be risk factors for eating disorders-related diseases. There is a positive correlation between food approach behaviors (food enjoyment, hunger, emotional overeating, and food responsiveness) and symptoms of food addiction (FA) (12) Selective or “picky” eating (13) and satiety response (14) are associated with avoidance/restrictive food intake disorder (ARFID).

The Behavioral Susceptibility Theory (BST) explains how genes and the environment interact in the development of overweight (15). Inherited appetitive traits interact with the food environment to influence obesity risk. Individuals with more “obesogenic” appetitive traits, like higher responsiveness to food and lower responsiveness to satiety cues, are more likely to overeat in the presence of highly palatable food (16). Importantly, emerging evidence indicates that these appetitive tendencies can be modified, suggesting that identifying and targeting specific appetite-related behaviors may be an effective strategy for weight management (17, 18).

Several psychometric instruments have been developed to assess eating-related traits, including the Dutch Eating Behavior Questionnaire (DEBQ) (19), the Three-Factor Eating Questionnaire (TFEQ) (20), and the Adult Eating Behavior Questionnaire (AEBQ) (21). Among them, the AEBQ is the most comprehensive tool for assessing self-reported appetitive traits in adults. It includes eight subscales—four food-approach traits (Hunger, Food Responsiveness, Emotional Over-eating, Enjoyment of Food) and four food-avoidance traits (Satiety Responsiveness, Emotional Under-eating, Food Fussiness, Slowness in Eating), which can provide accurate feedback on the characteristic responses to managing appetite, thereby reporting whether individuals need intervention to control weight and dietary habits (21). The AEBQ has been validated in multiple populations, including the UK (22), Australia (23), Canada (24), Mexico (25), Turkey (26), Portugal (27), Saudi Arabia (28), and China (9), and consistently demonstrates strong validity and reliability.

Despite its wide use, findings regarding the optimal factor structure of the AEBQ remain inconsistent. Studies from Western populations often support a seven-factor model excluding the Hunger subscale (22–24). In contrast, a Chinese validation study found the eight-factor model to perform better, but it did not evaluate a model excluding the Hunger subscale (9). These discrepancies suggest that the suitability of the Hunger subscale may vary across populations and should be examined in further research, calling for re-evaluation of the factor structure of the AEBQ in the Chinese context.

Given the cultural and behavioral differences in eating habits between Chinese and Western populations, it is essential to evaluate whether the AEBQ retains its psychometric robustness in a Chinese context. Understanding the structure and applicability of the AEBQ in China would facilitate cross-cultural comparisons and enhance the tool’s use in both research and intervention settings. Therefore, the present study aimed to provide comprehensive psychometric validation of the Adult Eating Behavior Questionnaire (AEBQ) among Chinese university students. Specifically, we sought to (1) confirm the factor structure and internal consistency of the AEBQ, (2) assess its construct, concurrent, and discriminant validity through confirmatory factor analysis, inter-subscale correlations, and associations with BMI, and (3) explore gender and BMI-related differences in appetitive traits. Findings from this study will offer robust evidence supporting the AEBQ’s applicability in Chinese populations and its potential for advancing research and interventions targeting obesity-related eating behaviors.

## 2 Materials and methods

### 2.1 Study design and participants

A cross-sectional study was designed to evaluate the psychometric properties of the Chinese version of the Adult Eating Behavior Questionnaire (AEBQ) among university students and examine the relationships between appetitive traits and body mass index (BMI). It took place in Nanjing, China, recruiting around 500 university students from Southeast University. Data was gathered through face-to-face questionnaires, available in both Chinese and English paper versions, which required approximately 5 min to complete.

### 2.2 Measures

#### 2.2.1 Sociodemographic questionnaire

Information on participants’ age, gender, height, weight, education (bachelor; master; Phd), and profession was collected through a sociodemographic questionnaire.

#### 2.2.2 Adult eating behavior questionnaire (AEBQ)

Appetitive traits, including food approach and food avoidance traits, were assessed using the AEBQ (21). The questionnaire has been validated in a group of adolescents aged 11 to 18 years (22) and in a group of young adults aged 17 to 24 years (29, 30), and can provide reliable data.

The AEBQ is a self-report questionnaire with 35 items, divided into two subscales: four “food approach” subscales and four “food avoidance” subscales. The four “food approach” subscales assess are following: (1) Hunger (H)-five items (e.g., “If my meal is delayed, I often feel dizzy”); (2) Food Responsiveness (FR) - four items (e.g., “When I see or smell my favorite food, it makes me want to eat it”); (3) Emotional Over-eating (EOE) - five items (e.g., “I eat more when I am upset”); (4) Enjoyment of Food (EF) - three items (e.g., “I look forward to mealtimes”). The food approach subscale defines behaviors involving craving for food (31). The four food avoidance subscales assess are following: (1) Satiety Responsiveness (SR) - four items (e.g., “If I had eaten a snack before dinner, I would not want to eat”); (2) Emotional Under-eating (EUE) - five items (e.g., “I eat less when I am annoyed”); (3) Food Fussiness (FF) - five items (e.g., “I often think I should not like it before I eat some kind of food”); (4) Slowness in Eating (SE) - four items (e.g., “During the meal, I eat more slowly”). The food avoidance subscale defines behaviors that involve staying away from food (31). The scores of each item were recorded on a five-point Likert scale, ranging from “1 = Strongly Disagree” to “5 = Strongly Agree.” The subscale scores were calculated based on the mean of the items within each subscale.

To ensure both linguistic and cultural equivalence of the Chinese version, a rigorous translation and cultural adaptation procedure was followed. Two bilingual Chinese nutrition experts independently translated the original English AEBQ into Chinese to ensure linguistic accuracy and cultural relevance. A native English-speaking researcher then performed a back-translation into English. The three translators compared the original and back-translated versions and reached consensus on any discrepancies. A key focus was cultural adaptation, for which an expert panel of two nutrition and one psychology researcher evaluated the items for conceptual equivalence, cultural appropriateness, and clarity. Subsequently, cognitive interviews were piloted with 25 students to assess the items’ comprehensibility, relevance, and clarity. Participant feedback confirmed that the translated version was highly understandable and culturally appropriate, requiring almost no further modification.

#### 2.2.3 Anthropometric characteristics

BMI was calculated using self-reported height and weight data. It is the most widely used indicator of obesity and is calculated as weight in kilograms divided by height in meters squared (kg/m2). According to the BMI classification criterion for the Chinese population (32), participants were divided into four groups: underweight (BMI < 18.5 kg/m2), normal weight (BMI: 18.5–23.9 kg/m2), overweight (BMI: 24.0–27.9 kg/m2), and obese (BMI ≥ 28.0 kg/m2). BMI provides a practical method for measuring obesity in population-level epidemiological studies that evaluate the health hazards linked to obesity. Additionally, it continues to serve as an indicator of obesity in most current epidemiological studies (33). Since self-reported data may exist with potential reporting bias, this limitation was acknowledged and considered when interpreting the results.

### 2.3 Statistical analysis

Before analysis, data were checked for missing values and outliers. Given the low proportion of missing data (<5%), the Expectation–Maximization (EM) algorithm was employed for imputation under the Missing at Random (MAR) assumption, thereby minimizing potential bias while preserving statistical power (34). The normality of the data distribution was tested by applying the Kolmogorov–Smirnov test. Continuous variables were described using the median and interquartile range (Median±IQR) due to non-normal distribution. Categorical variables were described using frequencies and corresponding percentages [n (%)]. Statistical comparisons were performed using the nonparametric Mann–Whitney U test and the Kruskal-Wallis H test.

Confirmatory factor analysis was conducted using Structural Equation Modeling (SEM), which tested the fit of data from three alternative models based on the findings of Hunot et al. (21). Model 1 included all original 35 items and 8 subscales; Model 2 included all 35 items and 7 subscales, combining Hunger and Food Responsiveness into a single subscale; and Model 3 included 30 items and 7 subscales, deleting the Hunger subscale (5 items). Model fit was assessed using the following indices: chi-square statistic (χ^2^), degrees of freedom (df), Comparative Fit Index (CFI), Tucker Lewis Index (TLI), and Root Mean Square Error of Approximation (RMSEA). When χ^2^/df < 3, CFI and TLI values>0.90 (35), and RMSEA ≤ 0.06 (36), it indicates that the model fits well. The parsimony of alternative models was assessed using Akaike’s information criteria (AIC) to select the best-fitting model, where smaller values indicated a more parsimonious model (37). Statistical significance was set at *p*-values <0.05.

Descriptive statistics and reliability evaluation were conducted on the AEBQ scale. Internal consistency of the AEBQ was assessed using Cronbach’s alpha (*α*), and Cronbach’s α greater than 0.70 was considered a good measure of consistency for each appetitive trait (38). McDonald’s omega coefficient (*ω*) was also calculated to eliminate potential errors in internal consistency estimation (39). Spearman correlation analysis was used to examine the relationships between the AEBQ scales, as well as the relationship between appetitive traits and BMI. Statistical analysis was performed using IBM SPSS V.27, and structural equation modeling was completed using IBM SPSS AMOS V.28.

## 3 Results

### 3.1 Demographic characteristics

Table 1 displays the descriptive characteristics of the university student sample in this study. A total of 546 participants were included in the final analysis, with a median age of 20.0 ± 3.0 years and a median body mass index (BMI) of 20.8 ± 3.8 kg/m^2^. Among them, 339 were female (62.1%) and 207 were male (37.9%). Based on BMI classification, 98 participants (17.9%) were underweight, 360 (65.9%) had normal weight, 69 (12.6%) were overweight, and 19 (3.5%) were obese.

**Table 1:** Descriptive characteristics of the sample of 546 Chinese university students in this study.

Characteristics	Total (*n* = 546)	Male (*n* = 207)	Female (*n* = 339)
Age (years)	20.0 ± 3.0	20.0 ± 3.0	19.0 ± 3.0
BMI(kg/m^2^)	20.8 ± 3.8	22.0 ± 3.7	20.2 ± 3.3
underweight (BMI < 18.5)	98 (17.9%)	22 (4.0%)	76 (13.9%)
normal weight (BMI: 18.5–23.9)	360 (65.9%)	133 (24.4%)	227 (41.5%)
overweight (BMI: 24–27.9)	69 (12.6%)	39 (7.1%)	30 (5.5%)
obese (BMI ≥ 28)	19 (3.5%)	13 (2.4%)	6 (1.1%)
Bachelor	492 (90.1%)	187 (34.2%)	305 (55.9%)
Master	37 (6.8%)	13 (2.4%)	24 (4.4%)
PhD	17 (3.1%)	7 (1.3%)	10 (1.8%)

Me±IQR or (*n*) %.

### 3.2 Confirmatory factor analysis

Table 2 displays the fit indices for the three AEBQ alternative models. The two other seven-factor models (Models 2 and 3) and the original eight-factor AEBQ (Model 1) were compared for goodness of fit using CFA. According to the study’s findings, all three models had a reasonably acceptable model fit (defined as *χ*^2^/df < 3.0, TLI and CFI values > 0.90, and RMSEA values ≤ 0.06) (35).

**Table 2:** Goodness of fit statistics of three models of the AEBQ evaluated via confirmatory factor analysis in the sample of 546 Chinese university students.

Model	Structure	*χ* ^2^	df	*χ*^2^/df	TLI	CFI	RMSEA(90%CI)	AIC
1	All original 35 items, 8 subscales	1,401.838	532	2.635	0.922	0.930	0.055(0.051, 0.058)	1,667.838
2	All original 35 items, 7 subscales, FR and H combined into one subscale	1,578.086	539	2.928	0.908	0.917	0.059(0.056, 0.063)	1,830.086
3	30 items, 7 subscales, H scale deleted (5 items)	1,078.881	384	2.810	0.931	0.939	0.058(0.054, 0.062)	1,300.881

FR, Food Responsiveness Scale, H, Hunger Scale; χ^2^ chi-square statistic, df, degrees of freedom, TLI, Tucker-Lewis index, CFI, comparative fit index, RMSEA, root mean square error of approximation, CI, confidence interval, AIC Akaike’s information criterion.

The original 35-item, 8-factor model (Model 1) outperformed significantly the 35-item, 7-factor model (Model 2) on all fit indices taken into consideration. Additionally, Model 1 was more parsimonious based on the AIC value. Despite not being directly comparable to Models 1 and 2 because of the different number of items, the 30-item, 7-factor model (Model 3) also demonstrated an overall good fit. Furthermore, the AIC values showed that when the Hunger subscale was eliminated from the analysis, the fit statistics greatly improved.

### 3.3 Descriptive statistics and internal consistency estimates for the AEBQ

The reliability estimates and scale scores for the AEBQ are presented in Table 3. In the current sample, all subscales showed good internal reliability with Cronbach’s *α* values >0.70 and McDonald’s *ω* values >0.70. The scale scores indicated that the scores of the food approach scales were relatively higher than those of the food avoidance scales.

**Table 3:** Descriptive statistics (Me±IQR) and internal consistency estimates (Cronbach’s *α* and McDonald’s *ω*) for the 8 factor AEBQ in the sample of 546 Chinese university students.

AEBQ subscales		Number of items	Me±IQR	Cronbach’s *α*	McDonald’s *ω*
Food approach subscales	Enjoyment of food	3	4.0 ± 1.3	0.906	0.908
	Emotional over-eating	5	2.6 ± 1.4	0.944	0.944
	Food responsiveness	5	3.0 ± 1.0	0.702	0.709
	Hunger	5	2.6 ± 1.0	0.754	0.759
Food avoidance subscales	Food fussiness	4	2.4 ± 1.0	0.821	0.820
	Emotional under-eating	5	3.0 ± 2.0	0.958	0.958
	Slowness in eating	4	2.5 ± 1.5	0.869	0.875
	Satiety responsiveness	4	2.8 ± 1.5	0.786	0.798

Me: median, IQR: interquartile range, the same applies below.

### 3.4 Descriptive analysis of the AEBQ subscales and the relationship between appetitive traits and BMI

The comparison of the AEBQ subscale scores between males and females in this study is presented in Table 4. The findings indicated that, with statistically significant differences, females reported higher scores on the measures of Enjoyment of Food, Emotional Over-Eating, Food Responsiveness, and Satiety Responsiveness compared to males, with *p*-values of *p* = 0.010, *p* = 0.013, *p* = 0.011, and *p* < 0.001, respectively. Additionally, their overall AEBQ scores also exceeded those of males (*p* < 0.001). This suggests that it seems females’ eating behavior is more susceptible to appetite.

**Table 4:** Comparison between AEBQ subscale scores for males and females in the sample of 546 Chinese university students.

Gender		EF	EOE	FR	H	FF	EUE	SE	SR	Total AEBQ
Male	Me	4.0	2.4	2.8	2.6	2.6	3.0	2.3	2.5	98.0
	IQR	1.3	1.0	1.0	1.0	1.0	2.0	1.5	1.0	18.8
Female	Me	4.3	2.8	3.0	2.6	2.4	3.0	2.5	3.0	102.0
	IQR	1.0	1.6	1.0	1.0	1.0	2.0	1.3	1.3	15.0
*Z*		2.580	2.492	2.555	1.798	−1.127	0.528	1.914	4.981	4.149
*p*-value*		0.010	0.013	0.011	0.072	0.260	0.597	0.056	<0.001	<0.001

EF, enjoyment of food; EOE, emotional over-Eating; FR, food responsiveness; H, hunger; FF, food fussiness; EUE, emotional under-Eating; SE, slowness in eating; SR, satiety responsiveness. *Mann–Whitney U test.

The correlations between the different subscales of AEBQ are listed in Table 5. The food approach scales showed positive correlations with each other and generally negative correlations with the food avoidance scales. Enjoyment of Food had a significant negative correlation with Food Fussiness, Emotional Under-Eating, and Satiety Responsiveness. Emotional Over-Eating had a significant negative correlation with Food Fussiness and Emotional Under-Eating, but no correlation with Slowness in Eating and Satiety Responsiveness.

**Table 5:** Correlations between the eight factor AEBQ subscales in the sample of 546 Chinese university students.

AEBQ subscales		Food approach subscales				Food avoidance subscales				Total AEBQ
		EF	EOE	FR	H	FF	EUE	SE	SR	
Food approach subscales	EF	1	0.229**	0.324**	0.158**	−0.358**	−0.139**	−0.078	−0.190**	0.164**
	EOE		1	0.246**	0.212**	−0.096*	−0.527**	0.023	−0.081	0.290**
	FR			1	0.506**	−0.046	−0.034	0.033	−0.034	0.539**
	H				1	0.042	−0.037	0.229**	0.118**	0.630**
Food avoidance subscales	FF					1	0.099*	0.025	0.225**	0.322**
	EUE						1	0.145**	0.221**	0.228**
	SE							1	0.401**	0.541**
	SR								1	0.501**

EF, enjoyment of food; EOE, emotional over-eating; FR, food responsiveness; H, hunger; FF, food fussiness; EUE, emotional under-eating; SE, slowness in eating; SR, satiety responsiveness. *p < 0.05; **p < 0.01.

Surprisingly, Food Responsiveness did not show any correlation with the food avoidance traits. Hunger had a positive correlation with Slowness in Eating and Satiety Responsiveness, but not with Food Fussiness and Emotional Under-Eating. The food avoidance scales also had positive correlations with each other, but Food Fussiness did not show a significant correlation with Slowness in Eating.

The correlations between appetitive traits and BMI are summarized in Table 6. Among the food approach subscales, Enjoyment of Food and Emotional Over-Eating were positively correlated with BMI (*p* < 0.05), whereas Food Responsiveness and Hunger showed no significant associations. Consistent with expectations, all food avoidance subscales (Food Fussiness, Emotional Under-Eating, Slowness in Eating, and Satiety Responsiveness) were negatively correlated with BMI (*p* < 0.05). The total AEBQ score was also significantly negatively correlated with BMI (*p* < 0.01). After adjusting for gender and age, Food Responsiveness became significantly positively associated with BMI, while the previously significant association between Food Fussiness and BMI was attenuated and became non-significant; other relationships remained unchanged.

**Table 6:** Correlations between the eight original AEBQ subscales and BMI in the sample of 546 Chinese university students.

Variable	Food approach subscales				Food avoidance subscales				Total AEBQ
	EF	EOE	FR	H	FF	EUE	SE	SR	
BMI	0.087*	0.147**	0.070	−0.078	−0.089*	−0.117**	−0.249**	−0.398**	−0.172**
BMI^a^	0.019*	0.192**	0.131*	−0.049	−0.069	−0.124*	−0.238**	−0.353**	−0.125*

EF, enjoyment of food; EOE, emotional over-eating; FR, food responsiveness; H, hunger; FF, food fussiness; EUE, emotional under-eating; SE, slowness in eating; SR, satiety responsiveness. *p < 0.05; **p < 0.01; ^a^analyses adjusted for gender and age.

Table 7 presents the results of intergroup comparisons of AEBQ subscale scores across BMI categories using the Kruskal–Wallis *H* test, followed by *post hoc* analyses. No significant differences were observed among the food approach subscales across different BMI categories (all *p* > 0.05), whereas all four food avoidance subscales showed significant differences (all *p* < 0.05). Participants in the underweight group scored significantly higher on the food avoidance subscales. Specifically, compared with overweight participants, underweight participants reported significantly higher scores on Emotional Under-Eating, Slowness in Eating, Satiety Responsiveness, and the total AEBQ score.

**Table 7:** Results of intergroup comparisons of AEBQ subscales scores across BMI categories using the Kruskal–Wallis *H* test and *post hoc* analysis.

AEBQ Subscales		I Underweight (n = 98)	II Normal weight (n = 360)	III Overweight (n = 69)	IV Obese (n = 19)	*H*	*p*-value	*Post-hoc*
Food avoidance subscales	EF	4.0 ± 1.7	4.0 ± 1.0	4.0 ± 1.3	4.0 ± 1.0	6.013	0.111	n.s.
	EOE	2.3 ± 1.2	2.6 ± 1.4	3.0 ± 2.0	3.0 ± 2.6	7.231	0.065	n.s.
	FR	3.0 ± 1.3	3.0 ± 1.0	3.0 ± 1.3	3.3 ± 1.0	4.264	0.234	n.s.
	H	2.8 ± 0.8	2.6 ± 1.0	2.4 ± 1.3	2.4 ± 0.6	4.738	0.192	n.s.
Food avoidance subscales	FF	2.8 ± 1.0	2.4 ± 1.2	2.4 ± 1.4	2.6 ± 1.0	10.292	0.016	I > II**
	EUE	3.3 ± 1.3	3.0 ± 2.0	2.8 ± 2.0	3.0 ± 2.0	10.106	0.018	I > III*
	SE	2.8 ± 1.3	2.5 ± 1.3	2.0 ± 1.5	2.0 ± 1.5	30.166	<0.001	I > II > III** I > IV**
	SR	3.3 ± 1.1	2.8 ± 1.3	2.3 ± 1.3	2.0 ± 1.0	72.729	<0.001	I > II > III** II > IV *
Total AEBQ		105.0 ± 16.5	100.5 ± 15.0	95.0 ± 17.5	100.0 ± 10.0	18.749	<0.001	I > III** I > II*

EF, enjoyment of food; EOE, emotional over-eating; FR, food responsiveness; H, hunger; FF, food fussiness; EUE, emotional under-eating; SE, slowness in eating; SR, satiety responsiveness. n.s, no significance;*p < 0.05; **p < 0.01.

## 4 Discussion

This study translated and validated the Chinese version of the AEBQ among Chinese university students, confirming its factorial structure, internal consistency, and associations between appetitive traits and BMI. The results demonstrate that the AEBQ is a reliable and valid tool for assessing appetitive traits in Chinese adults, supporting its applicability in future obesity-related research and intervention design.

This study employed CFA to compare three AEBQ models previously proposed in the literature and to determine the most appropriate structure for Chinese university students. Particularly, the analysis focused on whether the fit would be improved by combining the Hunger Scale with the Food Responsiveness Scale (Model 2) or removing the Hunger Scale from the questionnaire (Model 3). Based on the CFA goodness-of-fit metrics, it can be concluded that the model fit was relatively good for all three models (40), which is consistent with the test results of Mallan et al. in an Australian sample (23). Furthermore, removing the Hunger subscale yielded the best model fit, as indicated by improved fit indices (e.g., smaller AIC). Similar results were also found in validation studies involving Canadian adults (24), Mexican adults (25), and British adolescents (22). This suggests that the Hunger subscale may not perform well in these populations, possibly because hunger perception is influenced by situational and cultural factors. For instance, the satiety state of participants may alter individual responses to hunger-related questions (41). Hunger may be perceived as a transient physiological state rather than a stable appetitive trait (25).

This study further supports the association between specific appetitive traits and BMI in the Chinese adult population. Our findings differ from those reported by He et al. (9), who found no significant correlation between any food approach subscales and BMI in their sample from Zhejiang and Liaoning provinces. In contrast, we identified significant positive correlations of Emotional Over-eating (EOE) and Enjoyment of Food (EF) with BMI among adults in Jiangsu province. After adjusting for gender and age, Food Responsiveness (FR) also showed a positive association with BMI. Additionally, while He et al. observed no link between Emotional Under-eating (EUE) and BMI, our results revealed a significant negative association. These discrepancies may reflect regional differences in dietary habits and cultural environments across China. Our observations are nonetheless consistent with international studies using the AEBQ in various populations, such as Turkish adults (EOE, EF positively correlated with BMI) (26), Portuguese adolescents (EOE positively, SE and FF negatively correlated with BMI) (27), and Canadian adults (FR, EOE positively and EUE, SE negatively correlated with BMI) (24). Such cross-cultural parallels reinforce the robustness and applicability of the AEBQ as an assessment tool.

When comparing gender differences, the present study observed that females scored significantly higher than males on EF, EOE, FR, and SR, with higher total AEBQ scores. These findings are consistent with findings from previous populations, such as Polish adolescents (42) and UK adolescents (22), where females also showed stronger emotional and external eating tendencies. The underlying mechanisms may involve both behavioral and biological factors. Behaviorally, females tend to eat more slowly and chew more thoroughly (43), which enhances awareness of internal satiety cues and may partly explain their higher SR scores (44). From a neurobiological perspective, functional neuroimaging studies indicate that females exhibit heightened neural reactivity to food cues, particularly in craving- and taste-related brain regions like the anterior insula and orbitofrontal cortex, with enhanced responses under food deprivation (45, 46). Additionally, hormonal fluctuations across the menstrual cycle, such as increased progesterone during the luteal phase (47), may also amplify emotional eating tendencies in females (48).

This study has certain limitations. Firstly, the use of self-reported data for both the AEBQ responses and height/weight measurements introduces the potential for recall and social desirability biases, which may affect the accuracy of the assessed appetitive traits and BMI values (49). Future studies had better consider objective measurements of height and weight for BMI calculation. Secondly, the sample was primarily drawn from a university student population, which limits the generalizability of our findings to the broader Chinese adult population (50). Future research should validate the AEBQ in more diverse community-based and clinical samples. Additionally, the use of mean scores instead of factor scores for the AEBQ potentially has overlooked significant item weights within subscales. Lastly, as a cross-sectional design, this study cannot establish causality between appetitive traits and BMI. Prospective longitudinal studies are needed to elucidate the directionality of these associations. Tracking appetitive traits from infancy through adulthood, potentially by combining instruments such as the BEBQ (Baby Eating Behavior Questionnaire) (51), CEBQ (Children Eating Behavior Questionnaire) (52), and AEBQ, could enable early identification of at-risk individuals and inform targeted interventions for weight management.

Recent studies have explored pharmacological and psychological approaches that target appetitive traits to support weight management. For example, semaglutide (53), liraglutide (54), and the non-toxic bioactive peptide D3 (55) have shown potential in reducing appetite and body weight. The probiotic supplement LPR (*Lactobacillus rhamnosus* CGMCC1.3724) can improve satiety and decrease food cravings via the gut-brain axis (56). Moreover, Appetite-focused cognitive behavioral therapy (CBT-A) may be an effective treatment for overeating by helping individuals identify and respond to hunger and satiety cues, and restoring a normal diet (57). Cue-Exposure Treatment for Food (CET FOOD) was designed to reduce eating in response to food cues when full to reduce satiety responsiveness. It has been demonstrated to have feasibility, acceptability, and preliminary efficacy in children and adults (58). Further randomized clinical trials can use the AEBQ to quantify appetitive traits and monitor behavioral changes, thereby supporting objective evaluation of intervention efficacy and advancing the understanding of behavioral mechanisms underlying weight regulation.

In the future, the validated Chinese version of the AEBQ may serve as a valuable assessment tool for obesity prevention programs and clinical practice in China. At the public health level, it can be applied in large-scale population screening to identify individuals with high-risk appetitive traits, such as elevated food approach behaviors (e.g., Food Responsiveness, Emotional Over-Eating), thereby informing targeted nutrition education and behavioral interventions in schools and communities. In clinical settings, physicians and dietitians can use AEBQ results to gain a deeper understanding of the behavioral and psychological drivers underlying patients’ obesity. For example, patients with high scores in Emotional Over-Eating may benefit from psychological support and emotion-regulation strategies. By translating latent appetitive traits into quantifiable dimensions, the AEBQ helps advance a more personalized approach to weight management, informed by distinct behavioral phenotypes.

## 5 Conclusion

The current study affirms the reliability and validity of the AEBQ in gauging appetitive traits among Chinese university students, demonstrating its cultural applicability and highlighting cross-cultural consistencies and variations in eating behavior. As a comprehensive and convenient self-report measure, the AEBQ is expected to be valuable for evaluating a wide range of eating behaviors related to appetite, identifying obesity risk behaviors among adults, and conducting large-scale research in Chinese populations. The study also established that food approach traits (except for Hunger) were significantly and positively associated with BMI, whereas food avoidance traits (except for Food Fussiness) were significantly and negatively associated with BMI, suggesting the crucial role of appetite in weight development. However, given the cross-sectional nature of this study, causal inferences regarding the direction of these relationships cannot be established. Future research should prioritize prospective longitudinal designs to clarify the directionality of the association between appetitive traits and BMI. In parallel, the development of interventions specifically targeting appetitive traits may provide practical avenues for weight management and obesity treatment.

## Data Availability

The original contributions presented in the study are included in the article/supplementary material, further inquiries can be directed to the corresponding author.

## Glossary

AEBQ: Adult Eating Behavior Questionnaire
AIC: Akaike’s information criteria
ARFID: avoidance/restrictive food intake disorder
BEBQ: Baby Eating Behavior Questionnaire
BMI: body mass index
BST: Behavioral Susceptibility Theory
CBT-A: Appetite-focused cognitive behavioral therapy
CEBQ: Children Eating Behavior Questionnaire
CET FOOD: Cue-Exposure Treatment for Food
CFA: Confirmatory Factor Analysis
CFI: Comparative Fit Index
DEBQ: Dutch Eating Behavior Questionnaire
EF: Enjoyment of Food
EM: Expectation Maximization
EOE: Emotional Over-eating
EUE: Emotional Under-eating
FA: food addiction
FCQ: Food Choice Questionnaire
FF: Food Fussiness
FFQ: Food Frequency Questionnaire
FR: Food Responsiveness
H: Hunger
RMSEA: Root Mean Square Error of Approximation
ROC: Regulation of Cues
SE: Slowness in Eating
SR: Satiety Responsiveness
TFEQ: Three-Factor Eating Questionnaire
TLI: Tucker Lewis Index
